# The effects of plastic slatted floor and a deep- litter system on the growth performance of hybrid Pekin ducks

**DOI:** 10.5194/aab-64-1-2021

**Published:** 2021-01-04

**Authors:** Sabri Arda Eratalar

**Affiliations:** Poultry Science Department, Faculty of Agriculture, Bolu Abant İzzet Baysal University, Bolu, 14100, Turkey

## Abstract

This research was conducted to evaluate the effects of plastic slatted floors and a deep-litter system using wood shavings on the growth performance of current commercial hybrid Pekin ducks. A total of 96 Pekin ducks (Star 53) were reared for 42 d. Live weight, live-weight gain, feed consumption, feed conversion ratio, water consumption, and water / feed consumption ratio were investigated as the performance criteria. With the use of plastic slatted floors, the feed conversion rate dropped and the water / feed consumption ratio showed an incline (p<0.05). This is a very favourable result for the poultry industry and growers. The remaining parameters did not change by altering the ground system (p>0.05). Generally, it can be stated that plastic slatted floor use has advantages concerning the performance criteria of the feed consumption ratio and the water / feed consumption ratio in comparison to the deep-litter system. Furthermore, improvement in the feed conversion ratio is known to benefit the overall performance of poultry as well as having a positive economic impact. It should also be noted that as the birds grew, they were visually less stained, which is another important factor determining feather quality. However, this should be further investigated in future research.

## 1 Introduction

Compared to other poultry species and especially broilers, ducks are easier to grow, having greater adaptation ability to hot, cold, and humid environmental conditions (Wright, 2008; Holderread, 2011; HTEBooks, 2016). The demand for duck meat has increased during the last few decades, resulting in a total duck population of 2.1 billion, and meat production reached a total of 4 × 10${}^{x}$ t in 2010 (FAO, 2010) and is still increasing. Duck meat is the third most produced poultry meat in the USA and the fourth in the world (USDA, 2012). Pekin duck is an important poultry species for the European Union (EU) market. Pekin ducks are raised intensively in France, Germany, Hungary, Poland, and the UK, and extensively in some other European countries. For a long time, Pekin duck has been used in breeding studies with the aim of achieving better field performance with a better carcass yield and lower fat ratio than that of the parents (Ekarius, 2007; Wencek et al., 2012). Hybrid ducks that emerged as a result of years of breeding efforts were first given two types of diet and reared up to 7 weeks, reaching a live weight (LW) of 2.0–2.5 kg (Sainsbury, 1980; Dogan, 1987; Testik et al., 1988). For the first group of hybrid ducks, the slaughter weight was measured as 3195 g with a consumption of 8544 g of feed and a feed conversion ratio (FCR) of 2.675 (Leeson and Summers, 1980) and 3342 g (Knizetova et al., 1991), and more recently the FCR of hybrid ducks aged 42 d was reported as 2.5 and the slaughter weight as 3750 g (Holderread, 2011).

Generally, two types of diets are used when rearing ducks for meat production. The rearing period is considered to be around 7 to 9 weeks in countries demanding heavy birds and 6 to 8 weeks in recent commercial conditions. For the birds kept for 6 to 7 weeks, the first 2 weeks are known as the starter period, in which starter feed is given, and the second part is the growing period, in which the ducks are given grower–finisher feed. If the rearing period reaches 8 or 9 weeks, three types of feed are used: starter feed for the first 2 weeks, grower feed for the following 5–6 weeks, and the finisher diet for the last week or two (Knizetova et al., 1991).

As the performance of the hybrids has improved, the rearing conditions and systems have also evolved. Reared extensively a few decades ago, duck are kept in environmentally controlled poultry houses with a stocking density of three to seven ducks m${}^{-2}$ under commercial conditions similar to broilers and turkeys. With the growing demand for better performance and quality, new systems and better litter materials have been sought. Through years of research on poultry and especially broilers, the best litter material has been found as wood shavings (WSs) in terms of absorbing moisture and providing soft and warm bedding for the birds. For this reason, WSs and chopped straw are now very commonly utilized as duck litter worldwide (Dean and Sandhu, 2018).

The type of litter used in the poultry industry varies depending on availability and cost. However, it is important to decide on the appropriate material as litter due to the labour-extensive clean-out processes in the farm generating an additional expense for growers and industrialized companies as well as contributing to environmental pollution. The litter at the end of the production period is much more humid in duck production than in broiler and turkey production where the litter is also spread thinner in comparison (The Humane Society of the United States, 2008), resulting in a greater need of maintenance to keep the litter dry for healthy production. Thus, litter management and the material used constitute an important aspect of the production process, directly affecting the ducklings (Karcher et al., 2013). Lower-quality or poorly managed litter may even lead to injuries and disabilities among the ducklings (Broom, 2006). Lesions and injuries may develop from 7 d of age, depending on the litter type and material, type of drinker, feeder, feeding style, and genetics (Shepherd and Fairchild, 2010; Mayne, 2005; Mayne et al., 2007; Reiter et al., 1997; O'Driscoll and Broom, 2011; De Jong et al., 2012; Fraley et al., 2013).

In addition to WSs, straw, and rice hulls used as litter material in the standard deep-litter system, mesh wires and plastic slatted floor (PSF) are utilized as an alternative ground rearing system in duck production (The Humane Society of the United States, 2008). In previous research designed to investigate WSs and PSF as the litter and ground system, the ducks reared on slatted floor were reported to have cleaner feathers (Karcher et al., 2013), cleaner eyes, and higher live-weight gain (LWG) (Fraley et al., 2013).

In poultry production, growing, feeding, food safety, and welfare issues are followed very carefully by not only scientists from different disciplines but also consumers. However, despite the availability of several studies on layers and broilers (Eratalar, 2008; Webster et al., 1990; Lay et al., 2011), there is only limited research concerning the commercial duck growing conditions (Rodenburg et al., 2005; Jones and Dawkins, 2010). Several regulations and fundamental standards about duck rearing have been introduced in European countries (COE, 1999), but there is a lack of research data related to the litter material and ground systems specified in these regulations. Therefore, this study was conducted to obtain detailed information about the effects of using WS and PSF on the field performance of hybrid Pekin ducks and guide further research by providing a clue as to the current industrial stocking density differentiating the work from past research.

## 2 Material and methods

The experiment was conducted with 96 mixed-sex Grimaud Star 53, day old ducklings in a private research and development (R&D) rearing house located in the province of Bolu (40${}^{\circ }$46${}^{\prime }$44${}^{\prime \prime }$ N, 31${}^{\circ }$43${}^{\prime }$54${}^{\prime \prime }$ E), Turkey after obtaining special permission from the company and also with an ethical committee on animal use permit number of 2018–2019.

The ducklings were weighed and randomly placed in the trial pens in a random-parcels trial design, where the replicate numbers were calculated by power analysis software PASS 11 (Hintze, 2011) as four for each trial group and control group. The pens of the trial and control groups had an area of 4 m${}^{2}$ and a stocking density of three ducklings m${}^{-2}$. The R&D house contained eight pens in total with the dimensions of 2×2 m and was environmentally controlled by an automated system. As drinkers, four steel nipples (Gün-Tav – Flex 15, Turkey) placed on the watering pipe in each pen were connected to a water bin for each pen to easily measure the water consumed by the birds, and for each nipple, a water flow of 55–85 mL min${}^{-1}$ was provided. In addition, pan feeders (Gün-Tav Pan Feeder, Turkey) with a capacity of 10 kg of feed were used during the experiment. The ventilation system consisting of a tunnel fan with a flow of 4500 m${}^{3}$ h${}^{-1}$ (Bahçıvan BSM 400, Turkey) was used for cooling, and two minimum fans with a flow of 1100 m${}^{3}$ h${}^{-1}$ (Bahçıvan BPP 30 Turkey) were used for minimum ventilation. Automated electrical convection heaters (Flavel RI 3000M, Turkey) heated the rearing house throughout the experiment. The rearing house temperature of the growing period was 32${}^{\circ }$ at the beginning and gradually decreased (by 0.5${}^{\circ }$ every day), reaching 20${}^{\circ }$ on day 25, after which the temperature was maintained at $20{}^{\circ }\pm 2{}^{\circ }$ until the end of the rearing period. The automation control system used had been built specifically for the R&D rearing house to keep the climate inside the house stable during the whole production term.

The litter material was dry wood shavings of EU standard particle dimensions which were laid on the ground as 5 kg m${}^{-2}$. As the litter got wet it was changed for fresh litter material during the rearing period. The plastic slatted floor material was produced by a local company and had a depth of 8 cm, having 22×22 mm apertures (Vixpet Kümes Izgarası, Turkey) produced to EU standards.

The rearing period was divided into two: starter (the first 2 weeks) and growing (the last 4 weeks). A special formula from the Grimaud Star catalogue (Grimaud, 2016) was used for the feeding of the birds throughout the rearing period. The ducklings were given the starter feed for the first 2 weeks and the grower–finisher diet for 3 to 6 weeks ad libitum (Table 1). The feed used in the experiment was obtained from a private feed production company located in Bolu (Beypiliç© Yem).

**Table 1:** The nutritive and chemical values of the feed used in the experiment.

	Starter	Grower
	0–14 d	15 d slaughter
Metabolic energy, kcal kg${}^{-1}$	2900.00	3100.00
Crude protein, g kg${}^{-1}$	200.00	172.00
Crude cellulose, g kg${}^{-1}$	40.00	40.50
Crude fat, g kg${}^{-1}$	40.13	58.10
Crude ash, g kg${}^{-1}$	60.33	63.30
Lysine, g kg${}^{-1}$	10.00	8.00
Methionine, g kg${}^{-1}$	5.50	4.00
Calcium, g kg${}^{-1}$	100.00	90.00
Phosphorus, g kg${}^{-1}$	7.20	6.50
Sodium, g kg${}^{-1}$	1.60	1.70
Vitamin A, g kg${}^{-1}$	1.271	1.271
Vitamin D3, g kg${}^{-1}$	0.53	0.53
Manganese, mg kg${}^{-1}$	120.00	120.00
Zinc, mg kg${}^{-1}$	110.00	110.00
Copper, mg kg${}^{-1}$	16.00	16.00
Iodine, mg kg${}^{-1}$	1.50	1.50
Selenium, mg kg${}^{-1}$	0.30	0.30
NaCl, mg kg${}^{-1}$	0.44	0.42

In this research, LW, feed conversion (FC), and feed conversion ratio (FCR) were investigated as the primary performance criteria. The planning and implementation of the whole experiment was undertaken in accordance with the requirements of the Turkish National Committee for Experimental Animal Care (HAYEK). To obtain the actual LW values in the treatment groups, the poults were individually numbered by special plastic leg tags from 1 to 96. The LW measurements were taken by a precision (± 1 mg) scale (Radwag AS220R2, Poland) in the first 3 weeks and then by a normal (± 1 g) scale (TEM TNT 015D, Turkey) every week for the remainder of the experiment. Using the data obtained, the weekly LWG and the total live weight (TLW) per unit area were obtained at the end of the rearing period. In this experiment, the weekly FC was calculated by subtracting the amount of feed left in the feeder from the total weekly amount of feed placed in the feeder. From these data, the total and weekly FCR values, and the total FC and FC per duckling values were calculated. The mortality (M) was determined by recording the number of dead ducklings in the trial pens (if any), and based on these data, the daily, weekly, and total mortality rates were calculated. FCR was calculated with the following Eq. (1):

1 $$\text{Feed conversion ratio (FCR)}=\frac{\text{Feed consumed in kg (FC)}}{\text{Live weight in kg (LW)}}.$$

The trials were designed in random parcels and the statistical analyses of the data acquired from the experiment were performed by SPSS v. 22.0 (SPSS, 2013). The data obtained were first tested for homogeneity of variance. After confirming homogeneity (normality) by skewness and Kurtosis values, detailed statistical analyses were undertaken using a Shapiro–Wilk test. The statistical analyses of the treatments and the comparison of the mean values between the treatment groups were conducted using an analysis of variance (ANOVA) using the GLM procedure of SPSS one-way designs. The Tukey test was applied to determine whether the differences between the groups were statistically significant. P values less than 0.05 were considered to be statistically significant. The data obtained from the research were given as means ± standard error of the means (M ± SEM). The model used in the experiment is as follows:

2 Yij=μ+LMi+eij,

where Yij is the dependent variable, μ is the overall mean, LM${}_{i}$ is the effect of floor system (i: deep-litter system or plastic perforated floor system) and eij is the random error term.

## 3 Results

The data obtained from the experiment revealed that after the use of deep litter and PSF for 42 d of rearing period, the LW of the ducklings was 3314 ± 46 and 3450 ± 69 g, respectively. The LW of the treatment groups, which was similar within the initial weeks of the study, started to differ in later weeks and the numerical increase in the LW of the ducks reared on PSF became more prominent in the remainder of the rearing period and especially at the end (Table 2). Nevertheless, the difference between the LW of the treatment groups was not statistically significant (p>0.05).

To provide a better understanding of the LW parameter, the total live-weight gain (TLWG) values were investigated to evaluate LW values more elaborately, and the results were similar. In the first few weeks, the TLWG values of the two treatment groups seemed to be close to each other, but at later stages of the experiment, the differences turned out to be more in favour of the data obtained from the ducks reared on PSF, albeit with no statistical significance (p>0.05). Similarly, at the end of the rearing period, a visibly higher TLWG was achieved in the PSF group, but the results were, again, not statistically significant compared to the deep-litter group (p>0.05).

When the final TLWs calculated per unit area at the end of the rearing period were compared between the LW and PSF groups, the ducks reared on PSF were found to have higher values, but this difference was not statistically significant (p>0.05).

**Table 2:** The effects of PSF and WSs on the field performance of Pekin ducks (mean ± SEM).

	Litter material		
	Wood shavings	P. slatted floor	p values
Live weight, g per duckling per compartment			
Start${}^{*}$	54.50	54.50	
Second week	769 ± 13	734 ± 19	0.120
Fourth week	1584 ± 81	1564 ± 85	0.866
Sixth week	3314 ± 46	3450 ± 69	0.104
Total live-weight gain, g d${}^{-1}$			
Second week	51 ± 1	49 ± 1	0.120
Fourth week	55 ± 3	54 ± 3	0.866
Sixth week	78 ± 1	81 ± 2	0.104
Total live weight, kg m${}^{-2}$			
	9.94 ± 0.07	10.35 ± 0.17	0.069

${}^{*}$ Ducklings were weighed en masse at start.

Considering the effects of PSF and deep litter on FC and FCR, the total feed consumption (TFC) values were similar (Table 3); however, the differences between the treatment groups in terms of FC were not statistically significant (p>0.05). In contrast, the FCR values decreased visibly with the use of PSF as an alternative rearing ground system compared to deep litter (WS) through the rearing period. The difference in FCR between the two treatment groups was statistically significant at the slaughter age of 42 d (p<0.05).

**Table 3:** The effects of PSF and WSs on feed consumption and FCR of Pekin ducks (mean ± SEM).

	Litter material		
	Wood shavings	P. slatted floor	p values
Total feed consumption, g per duckling			
Second week${}^{*}$	1167	1167	
Fourth week	2924 ± 69	2807 ± 51	0.220
Sixth week	6160 ± 83	6039 ± 92	0.369
Feed conversion ratio (FCR)			
Second week	1.519 ± 0.032	1.596 ± 0.058	0.291
Fourth week	1.847 ± 0.037	1.813 ± 0.093	0.739
Sixth week	1.859 ± 0.020${}^{a}$	1.751 ± 0.022${}^{b}$	0.011

${}^{a,b}$ The different superscript letters on the same line indicate statistical significance (p<0.05). ${}^{*}$ The feed consumption for the first week was collected en masse.

When the water consumption (WC) and water feed${}^{-1}$ consumption ratio (WFCR) data of the experiment were analysed, it was determined that the weekly WC values per duckling were similar between the treatment groups (Table 3). However, the WC differences between the two groups were not statistically significant (p>0.05). In contrast, WFCRs started to increase after the fourth week in the PSF group, and the difference between the two groups became statistically significant at 6 weeks (p<0.05). This is considered to have been caused by the decreasing FCR, not the change in the amount of WC.

Water consumption data and WFCRs were not affected by the flooring systems except in the sixth week for WFCRs being a benefit for PSF as seen in Table 4.

**Table 4:** The effects of PSF and WSs on water consumption water / feed consumption ratio (mean ± SEM).

	Litter material		
	Wood shavings	P. slatted floor	p values
Water consumption, mL per duckling			
Second week	156 ± 3	152 ± 10	0.477
Fourth week	297 ± 4	297 ± 21	0.950
Sixth week	441 ± 8	469 ± 12	0.099
Water/feed consumption ratio			
Second week	1.875 ± 0.034	1.821 ± 0.062	0.477
Fourth week	2.849 ± 0.084	2.952 ± 0.057	0.351
Sixth week	3.010 ± 0.088${}^{a}$	3.259 ± 0.042${}^{b}$	0.041

${}^{a,b}$ The different superscript letters on the same line indicate statistical significance (p<0.05).

These parameters are currently used as performance criteria by the integrated companies where no experiment was found to compare our findings. The increase in WC / FC ratio in PSF in the sixth week is thought to have emerged from the decrease in FCR at the same age. This finding is beneficial for PSF, also lowering feeding and overall costs.

## 4 Discussion

The LW values obtained from the research were in line with some of the previous studies (Kinizetova et al., 1991), higher compared to the findings of other researchers (Sainsbury, 1980; Dogan, 1987; Testik et al., 1988), and lower than one report in a book (Holderread, 2011). In the current study, the LW values of the treatment groups became more prominent closer to the end of the rearing period, especially in the last week, when the LW values of the ducks reared on PSF also differed significantly statistically compared to the deep-litter group (p>0.05). In contrast, Fraley et al. (2013) reported similar LWG values for the treatment groups, including that those from PSF were higher than in our findings. This is thought to result from the differences in the rearing periods of these two studies.

The FC values obtained from the experiment were lower than the data presented by some researchers (Leeson and Summers, 1980; Holderread, 2011). Our FCR values were lower than those reported by other researchers (Sainsbury, 1980; Leeson and Summers, 1980; Dogan, 1987; Testik et al., 1988; Kinizetova et al., 1991; Holderread, 2011), and furthermore the use of PSF positively affected FCR (p<0.05). This may be explained by further behavioural experimentation regarding how birds tend to consume feed as they may have eaten the feed in the feeder bins rather than eating on the ground after taking from the feeders, resulting in less feed spoilage.

Our WC data were consistent with the findings of other publications (HTEBooks, 2016), and the use of PSF had a positive effect on WFCR (p<0.05).

Considering the overall results of the experiment, the use of PSF did not affect any of the field performance criteria but had a significant positive effect on FCR and WFCR. Besides, similar to the report of Karcher et al. (2013), the feathers, eyes, and generally the bodies of the ducks reared on PSF were observed to be cleaner, but we did not perform any statistical analyses concerning these parameters. Therefore, it can be stated that by replacing WS with PSF as an alternative rearing floor system, ducks with cleaner feathers can be obtained. However, this statement is only based on observation of the cleaner feathers, bills, and feet of the birds and the water pipelines in the PSF pens compared to the deep-litter group. The litter materials and floor systems should be taken into consideration in more detailed research to shed light on this subject and achieve cleaner and thus probably healthier birds and better field performance.

## 5 Conclusions

As a result, the use of PSF did not affect most of the field performance criteria but had a significant positive effect on FCR and WFCR, which should be significantly beneficial in industrial production and the overall economy of the sector.

Based on the results of the current research, we consider that PSF presents an advantageous and practically usable floor system as an alternative to deep litter with better FCR and WFCR values also decreasing workmanship visually; its use should be researched in more detail by researchers in the future regarding economics, behaviour, animal welfare aspects, and litter quality.

## Supplement

10.5194/aab-64-1-2021-supplement The supplement related to this article is available online at: https://doi.org/10.5194/aab-64-1-2021-supplement.

## Data Availability

The underlying research data can be obtained from the corresponding author via e-mail.
