# Supplementary material for: The effects of plastic slatted floor and a deep- litter system on the growth performance of hybrid Pekin ducks
Source: Arch Anim Breed. 2021 Jan 4;64(1):1–6. doi: 10.5194/aab-64-1-2021 (PMC8162238; doi:10.5194/aab-64-1-2021)
Supplement: The supplement related to this article is available online at: https://doi.org/10.5194/aab-64-1-2021-supplement. [file aab-64-1-supplement.pdf]

Supplement of Arch. Anim. Breed., 64, 1–6, 2021
https://doi.org/10.5194/aab-64-1-2021-supplement
© Author(s) 2021. This work is distributed under
the Creative Commons Attribution 4.0 License.

Open Access
Archives Animal Breeding

Supplement of

The effects of plastic slatted floor and a deep-litter system on the growth performance of hybrid Pekin ducks

Sabri Arda Eratalar

Correspondence to: Sabri Arda Eratalar (ardaeratalar@ibu.edu.tr)

The copyright of individual parts of the supplement might differ from the CC BY 4.0 License.

Tables

Table 1. The nutritive and chemical values of the feed used in the experiment.

	**Starter** 0-14 days	**Grower** 15 days-Slaughter
Metabolic energy kcal kg^{-1}	2,900.00	3,100.00
Crude protein, g kg^{-1}	200.00	172.00
Crude cellulose, g kg^{-1}	40.00	40.50
Crude fat, g kg^{-1}	40.13	58.10
Crude ash, g kg^{-1}	60.33	63.30
Lysine, g kg^{-1}	10.00	8.00
Methionine, g kg^{-1}	5.50	4.00
Calcium, g kg^{-1}	100.00	90.00
Phosphorus, g kg^{-1}	7.20	6.50
Sodium, g kg^{-1}	1.60	1.70
Vitamin A, g kg^{-1}	1.271	1.271
Vitamin D3, g kg^{-1}	0.53	0.53
Manganese, mg kg^{-1}	120.00	120.00
Zinc, mg kg^{-1}	110.00	110.00
Cupper, mg kg^{-1}	16.00	16.00
Iodine, mg kg^{-1}	1.50	1.50
Selenium, mg kg^{-1}	0.30	0.30
NaCl, mg kg^{-1}	0,44	0,42

Formula 1. The FCR calculation formula (Anonymous, 2012).

$$\text{Feed conversion ratio (FCR)} = \frac{\text{Feed consumed in kg (FC)}}{\text{Live weight in kg (LW)}}$$

Table 2. The effects of PSF and WS on the field performance of Pekin ducks (Mean ± SEM).

	**Litter Material**		
	**Wood Shavings**	**P. Slatted Floor**	**p-values**
**Live Weight, g/duckling/compartment**			
Start*	54.50	54.50	
2nd Week	769 ± 13	734 ± 19	0.120
4th Week	1,584 ± 81	1,564 ± 85	0.866
6th Week	3,314 ± 46	3,450 ± 69	0.104
**Total Live Weight Gain, g/day**			
2. Week	51 ± 1	49 ± 1	0.120
4. Week	55 ± 3	54 ± 3	0.866
6. Week	78 ± 1	81 ± 2	0.104
**Total Live Weight, kg/m²**			
	9.94 ± 0.07	10.35 ± 0.17	0.069

* Ducklings were weighed en masse at start.

Table 3. The effects of PSF and WS on feed consumption and FCR of Pekin ducks (Mean ± SEM).

	Litter Material		
	**Wood Shavings**	**P. Slatted Floor**	**p-values**
**Total Feed Consumption, g/duckling**			
2. Week*	1167	1167	
4. Week	2,924 ± 69	2,807 ± 51	0.220
6. Week	6,160 ± 83	6,039 ± 92	0.369
**Feed Conversion Ratio (FCR)**			
2. Week	1.519 ± 0.032	1.596 ± 0.058	0.291
4. Week	1.847 ± 0.037	1.813 ± 0.093	0.739
6. Week	1.859 ± 0.020[a]	1.751 ± 0.022[b]	0.011

[ab] The different superscript letters on the same line indicate statistical significance ($p < 0.05$).

* The feed consumption for the first week was collected en masse.

Table 4. The effects of PSF and WS on water consumption water/feed consumption ratio (Mean ± SEM).

	Litter Material		
	**Wood Shavings**	**P. Slatted Floor**	**p-values**
**Water Consumption, ml/duckling**			
2. Week	156 ± 3	152 ± 10	0.477
4. Week	297 ± 4	297 ± 21	0.950
6. Week	441 ± 8	469 ± 12	0.099
**Water / Feed Consumption Ratio**			
2. Week	1.875 ± 0.034	1.821 ± 0.062	0.477
4. Week	2.849 ± 0.084	2.952 ± 0.057	0.351
6. Week	3.010 ± 0.088[a]	3.259 ± 0.042[b]	0.041

[ab] The different superscript letters on the same line indicate statistical significance ($p < 0.05$).
